# Protein Structure, Evolution and Regulation of CER3, a Core Enzyme Partitioning Carbon into Two Specific Wax Biosynthetic Pathways

**DOI:** 10.3390/biology15161437

**Published:** 2026-08-20

**Authors:** Qingqing Niu, Limei Liu, Chang Liu, Shiyou Lü, Hui Zhang

**Affiliations:** 1 State Key Laboratory of Biocatalysis and Enzyme Engineering, School of Life Sciences, Hubei University, Wuhan 430062, China; 2 Hubei Hongshan Laboratory, Wuhan 430074, China

**Keywords:** cuticular wax, ECERIFERUM3 (CER3), evolution trajectory, regulation

## Abstract

Cuticular wax is a critical component of the plant cuticle, playing an indispensable role in plant growth, development, and defense against environmental stresses. ECERIFERUM3 (CER3), a very-long-chain fatty acyl-CoA reductase, serves as a pivotal hub channeling carbon resources into the alkane- and alcohol-forming pathways respectively. Here, we systematically characterized the protein structural features and evolutionary patterns of CER3. We also comprehensively reviewed the multiple regulatory mechanisms governing CER3, encompassing transcriptional, post-transcriptional, translational, and epigenetic modifications, with particular emphasis on the definitive finding that CER3 modulates cuticular wax biosynthetic flux by assembling distinct protein complexes. Finally, existing knowledge gaps and future research directions are also discussed.

## Simple Summary

CER3 is a central metabolic hub in plant wax biosynthesis, channeling carbon flux toward cuticular components and modulating epidermal barrier properties. Structural and evolutionary analyses reveal conserved architectural features of CER3 across plant lineages. Its activity is orchestrated at multiple regulatory levels: transcription, RNA surveillance, epigenetics, and ubiquitin-mediated turnover. CER3 also engages in dynamic protein complexes with CER1, SOH1, and CYP96A4 to balance alkane and alcohol pathways. Integrating these perspectives, this Review positions CER3 as a pivotal coordinator linking wax metabolism with environmental stress adaptation, offering insights for improving crop stress tolerance.

## 1. Introduction

The plant cuticle is a hydrophobic lipid barrier covering the aerial parts of land plants [1], playing a key role in coping with drought, low temperature, and pathogen infection by reducing non-stomatal water loss, reflecting ultraviolet radiation, blocking pathogen invasion, and maintaining epidermal structural integrity [2,3,4,5]. As an important constituent of the cuticle, waxes are either embedded within the scaffold structure formed by cutin or deposited on the surface to form various crystalline structures [6,7,8]. These wax components include both aliphatic compounds, primarily very-long-chain fatty acids (VLCFAs) and their derivatives, and aromatic compounds, which together exhibit species-specific distributions [9,10]. In response to drought, extreme temperatures, high light intensity, and other environmental challenges, the composition and relative abundance of wax constituents undergo dynamic changes accordingly [11,12].

Their synthesis initiates with de novo fatty acid synthesis of C16/C18 fatty acids in plastids, followed by elongation to VLCFAs catalyzed by the endoplasmic reticulum-localized fatty acid elongase complex consisting of 3-ketoacyl-CoA synthase (KCS), 3-ketoacyl-CoA reductase (KCR), 3-hydroxyacyl-CoA dehydratase (HCD) and enoyl-CoA reductase (ECR), and subsequent conversion to wax precursors via either the alkane- or the alcohol-forming pathway [13,14,15,16,17,18]. The synthesized waxes are transported to the cuticle via ATP-binding cassette G (ABCG) transporters and glycosylphosphatidylinositol-anchored lipid transfer proteins [19,20,21]. ECERIFERUM3 (CER3) encodes a very-long-chain fatty acyl-CoA reductase that catalyzes the reduction of VLC acyl-CoA to fatty aldehydes, which participate in both pathways [22]. Early mutant analyses mapped CER3 to chromosome 5, but it was erroneously annotated as an E3 ubiquitin ligase [23]. It was not known until Rowland and Colleagues corrected this error, confirming that CER3 (At5g57800) is allelic to WAX2/YRE/FLP1 [24]. Subsequently, Bernard identified CER3 as a key alkane-forming enzyme at the molecular level using a yeast heterologous expression system [22]. A recent study further revealed that CER3 also participates in the alcohol-forming pathway; it interacts with the aldehyde reductase SMART-OH1 (SOH1), thereby channeling carbon flux into alcohol biosynthesis [25].

Although the core function of CER3 is well established, existing research remains fragmented, lacking systematic integration of protein structure, evolutionary patterns, and multi-layered regulatory networks. To address this gap, we first analyzed the structural features of CER3 and reconstructed its evolutionary trajectory from lower to higher plants. Cuticular wax deposition is regulated by both internal factors, such as developmental stage, organ type, and hormones, and external factors, including biotic and abiotic stresses [26,27,28]. The spatially and temporally variable deposition patterns of cuticular wax are intimately linked to the differential expression profiles of wax biosynthetic genes. As a key enzyme in aliphatic wax biosynthesis, CER3 is subject to multifaceted regulation by diverse regulators, including transcription factors, E3 ligases, epigenetic modifiers, and interacting protein partners, etc. Accordingly, we comprehensively elucidated the molecular mechanisms underlying CER3 regulation as well. While previous reviews have comprehensively summarized the biochemical functions, regulatory mechanisms, and stress responses of CER1 and CER3 in model plants, and while the recent CER3–SOH1 study has elegantly elucidated the core shunt mechanism in Arabidopsis cuticular wax biosynthesis, an evolutionary perspective tracing CER3 across the full breadth of land-plant lineages has been lacking. In this review, we combine a systematic literature synthesis with phylogenetic analysis of CER3 orthologs across 43 representative species—from algae to angiosperms—to reconstruct the origin, duplication history, and lineage-specific diversification of CER3. By integrating functional findings from the literature with our phylogenomic inference, we propose an evolutionary model for CER3 functional specialization that complements existing mechanistic and regulatory frameworks. Taken together, this review provides deeper insights into cuticular wax biosynthesis, laying a theoretical foundation for genetic improvement of crop quality and postharvest preservation.

## 2. Protein Structure and Evolutionary Diversification of CER3 Across the Plant Kingdom

### 2.1. Protein Structure

**Figure 1:**
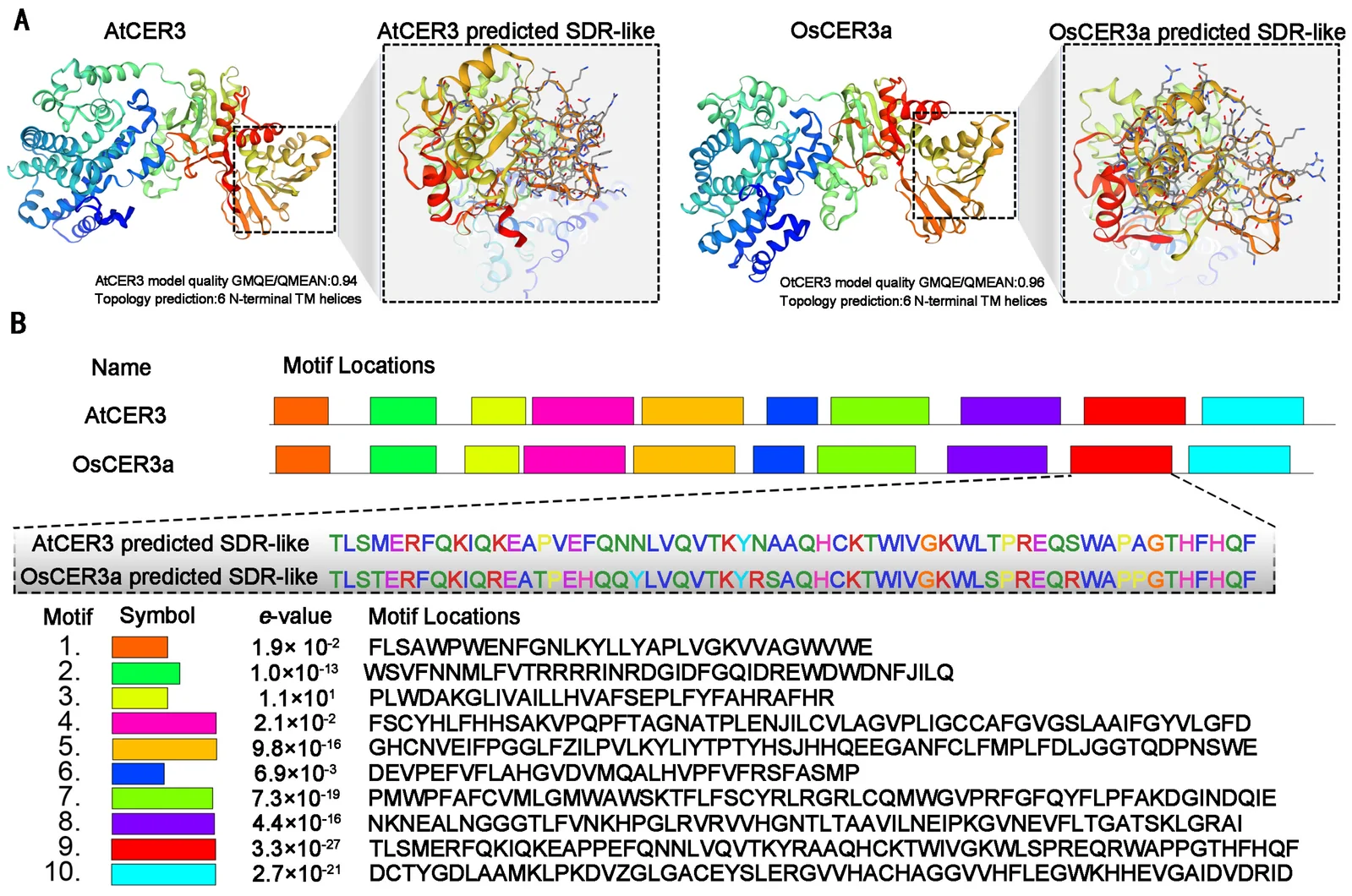
Structural motifs of Arabidopsis and rice CER3 proteins. (**A**) Predicted three-dimensional structures of AtCER3 and OsCER3a generated using SWISS-MODEL-based modeling (templates: AF-Q8H1Z0-F1 and AF-A2Z1F5-F1; GMQE = 0.94 and 0.96, respectively; Ramachandran favored: 97.78% and 89.84%). The overall protein architecture is shown on the left, while the putative SDR-like region embedded within the C-terminal WAX2 domain is enlarged on the right. The conserved folding patterns and spatially similar active-site configurations suggest a high degree of structural conservation between dicot and monocot CER3 proteins. Transmembrane topology was predicted using DeepTMHMM. Both AtCER3 and OsCER3a were predicted to contain six N-terminal transmembrane helices (AtCER3: residues 44–53, 55–64, 95–106, 128–146, 180–189, and 191–200; OsCER3a: residues 44–53, 55–64, 95–107, 125–142, 175–184, and 186–195). (**B**) Conserved motif composition of AtCER3 and OsCER3a identified using MEME analysis (v5.5.7; zoops mode; 10 motifs, width 32–60 aa; Motif 8 E-value = 3.0 × 10^−92^, Motif 9 E-value = 1.0 × 10^−77^). The ten conserved motifs (Motifs 1–10) are color-coded as follows: Motif 1 (blue), Motif 2 (cyan), Motif 3 (green), Motif 4 (yellow-green), Motif 5 (yellow), Motif 6 (orange), Motif 7 (red-orange), Motif 8 (purple), Motif 9 (red), and Motif 10 (pink), with their sequences, symbols, and E-values listed in the table. Ten conserved motifs distinguished by different colors were identified in both AtCER3 and OsCER3a proteins. Notably, the predicted SDR-like cofactor-binding region identified in this study is embedded within Motifs 8–9 (highlighted in purple and red), which contain the characteristic sequence signatures for nucleotide association. Note: Structural predictions and functional assignments presented here are computational in nature and await experimental validation.

CER3 possesses a conserved two-domain structure, with an N-terminal ERG3/FAH domain and a C-terminal WAX2 domain [29,30]. The following structural descriptions are based on computational predictions and in silico domain annotations; they have not been experimentally validated and should be interpreted with appropriate caution. The ERG3/FAH domain in the N-terminal region harbors a highly conserved histidine-rich motif (HX_3_H + HX_2_HH + HX_2_HH), which represents a characteristic signature of membrane-bound diiron enzymes involved in oxidation-reduction reactions [8,31]. This motif commonly presents in sterol desaturases, alkane hydroxylases and other integral membrane oxidoreductases [32,33]. The C-terminal WAX2 domain is indispensable for CER3 activity, as its disruption severely compromises cuticular wax accumulation in plants [24,34]. Notably, within the WAX2 domain, CER3 contains a signature motif characteristic of the short-chain dehydrogenase/reductase (SDR) superfamily, which adopts a conserved three-dimensional Rossmann fold structure [8,35]. According to InterPro domain prediction (SSF51735), this putative NAD(P)-binding module spans amino acid residues 456–524 in AtCER3 and corresponds to a conserved cofactor-binding region commonly found in members of the SDR superfamily, as bioinformatically predicted. Structural comparison showed that this predicted SDR-like region occupies a highly conserved spatial position in both Arabidopsis AtCER3 and rice OsCER3a, suggesting strong evolutionary conservation of its structural organization (Figure 1). Given that CER3 functions as a VLC acyl-CoA to fatty aldehydes, this conserved SDR-like module may provide the structural basis for NAD(P)H-dependent electron transfer during aldehyde formation. Motif analysis further corroborated the structural conservation of CER3 proteins. Ten highly conserved motifs were identified in both AtCER3 and OsCER3a, among which Motifs 1–5 are located in the N-terminal ERG3/FAH domain, whereas Motifs 6–10 are clustered within the C-terminal WAX2 domain (Figure 1B). Sequence alignment revealed that the predicted SDR-like module falls within the region of Motif 9, rather than being embedded within any single conserved motif, which is based on bioinformatic predictions and domain annotations rather than experimentally confirmed structural features. Despite this spatial separation, the structural integrity of the WAX2 domain, encompassing both the clustered conserved motifs and the intervening predicted SDR-like module that was predicted by computational analysis without experimental verification, appears to be essential for its function. The WAX2 domain, together with its conserved SDR-like module, exhibits markedly higher sequence conservation than the N-terminal region across land plants, a pattern consistently observed in phylogenetic analyses of 146 representative species [30]. Similar conservation of WAX2-domain conservation has also been observed in tomato, eucalyptus, and other angiosperms, supporting its evolutionary stability across diverse lineages [28,36]. The functional importance of this region is further supported by severe reductions in cuticular wax accumulation, reaching approximately 80% in Arabidopsis cer3 mutants and the rice wax-deficient mutant *wsl2* [24,37]. In the rice *wsl2* mutant, a single-nucleotide substitution disrupts splicing of the sixth intron, resulting in a premature stop codon and a truncated protein [37]. In Arabidopsis, the *cer3-1* and *cer3-4* mutations affect conserved regions of the WAX2/GL1-like protein, leading to impaired cuticular wax biosynthesis, although neither mutation directly disrupts predicted catalytic histidine-rich motifs or other conserved functional residues [24]. Collectively, these findings suggest that the WAX2 domain constitutes the principal functional core of CER3 proteins and that its sequence has been highly conserved throughout plant evolution.

CER3 and CER1 originated from an ancient gene duplication event in land plant evolution, followed by strong functional divergence [29,30]. Both proteins retain a similar domain architecture consisting of an N-terminal ERG3/FAH domain and a C-terminal WAX2 domain, supporting their common evolutionary origin. However, substantial sequence divergence has occurred following duplication, resulting in marked functional specialization. CER1 functions as a key component of the aldehyde decarbonylation complex responsible for alkane production, whereas CER3 primarily catalyzes the reduction of VLC acyl-CoAs to aldehydes. Although the ERG3/FAH domain remains highly conserved in CER3, its precise biochemical function remains elusive and may extend beyond direct catalysis to encompass structural stabilization or mediating protein–protein interactions within the alkane-forming complex [22]. In contrast, the predicted SDR-like module embedded within the WAX2 domain is well conserved in CER3 proteins, but appears highly degenerated in CER1 homologs, suggesting that this region may represent a key molecular feature underlying the functional divergence between CER1 and CER3 [22,30,38]. Collectively, current structural, motif, and evolutionary analyses support the view that the WAX2 domain, particularly its conserved predicted SDR-like module that was predicted by computational analysis, constitutes the primary functional region of CER3 proteins. Nevertheless, the molecular mechanisms through which this domain coordinates cofactor binding, substrate recognition, and aldehyde production remain largely unknown and warrant further investigation. Critical experimental validations, including cofactor binding tests, catalytic activity assays and mutational analyses, are lacking.

### 2.2. Evolutionary Trajectory and Functional Diversification of CER3 in the Plant Kingdom

**Figure 2:**
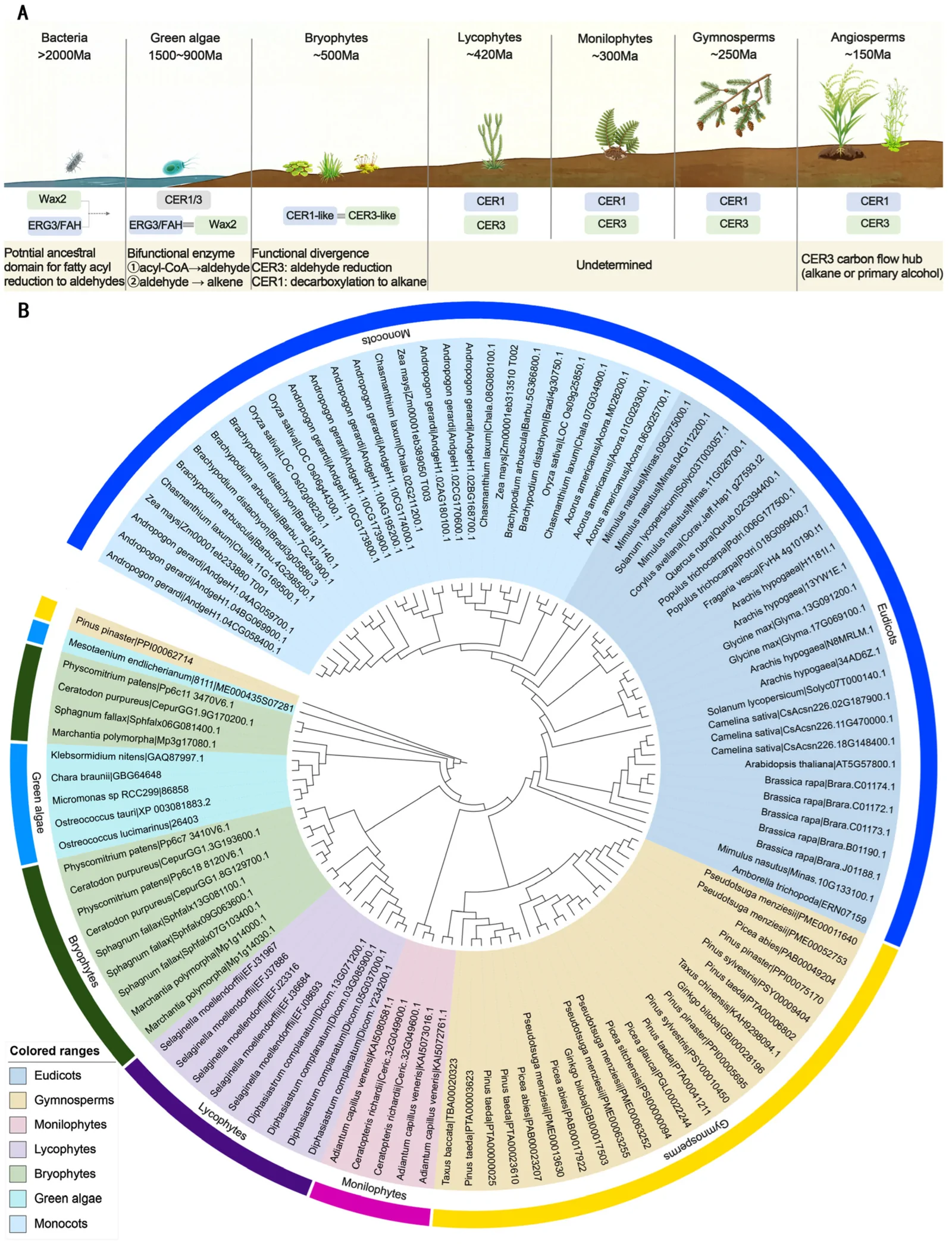
Phylogenetic tree of CER3 homologs and their evolutionary history. (**A**) The putative evolutionary trajectory of CER3 functional divergence is proposed to have begun from early land plants to seed plants, with key evolutionary nodes annotated for potential functional innovations or divergence events. (**B**) An unrooted maximum likelihood (ML) phylogenetic tree constructed from 113 CER3 orthologous protein sequences across 43 plant species. This tree contains only CER3 homologs; CER1 sequences were excluded during the manual filtration step. Sequences were retrieved from Phytozome (https://phytozome-next.jgi.doe.gov/, accessed on 27 May 2025), NCBI Genome (https://www.ncbi.nlm.nih.gov/datasets/genome/, accessed on 28 May 2025), Ensembl Plants (http://plants.ensembl.org/index.html, accessed on 29 May 2025), Joint Genome Institute (JGI) Genome Portal (https://genome.jgi.doe.gov/portal/, accessed on 28 May 2025), and Gymno PLAZA 1.0 (https://ftp.psb.ugent.be/pub/plaza/plaza_gymno_01/, accessed on 6 June 2026). Homologous sequences were initially identified by BLAST (2.17.0) using the Arabidopsis thaliana CER3 protein (AT5G57800) as the query, followed by profile HMM-based verification and Pfam domain annotation. Only sequences containing the PF12076 (WAX2) domain were retained, and the longest isoform was selected for each gene. The WAX2 domain sequences were aligned using MAFFT (v7.505) with the E-INS-i strategy (—genafpair—maxiterate 1000), and poorly aligned regions were trimmed with TrimAl (v1.4.rev22) using the -automated1 mode. The maximum-likelihood phylogeny was reconstructed using IQ-TREE2 (v2.2.2.7), with LG+G4 selected as the best-fit substitution model by ModelFinder according to the Bayesian Information Criterion (BIC). Branch supports were assessed with 1000 ultrafast bootstrap (UFBoot) replicates and 1000 SH-aLRT replicates. The species range spans from algae to angiosperms, with different colors representing distinct plant evolutionary lineages. The unrooted topology illustrates the clustering relationships and sequence affinities among CER3 orthologs across major plant lineages but does not, by itself, infer the direction of evolution or absolute divergence times (e.g., 450–500 Ma). The proposed evolutionary directions and timing shown in (**A**) are literature-based hypotheses integrated from references [30,38], rather than direct outputs of the phylogenetic tree in (**B**). Bootstrap support values (≥70%) are indicated at the nodes, and values below 70% are not displayed. The complete tree file with all detailed bootstrap and SH-aLRT support values is provided in Supplementary Table S1.

The evolutionary trajectory of CER3 can be traced through key stages of plant evolution. In bacteria and archaea, the WAX2 domain exists independently as the ancestral precursor. In green algae, a bifunctional CER1/3 enzyme arose via fusion of WAX2 and ERG3/FAH domains, enabling acyl-CoA to aldehyde and then to hydrocarbons. In bryophytes, gene duplication led to CER1 and CER3 divergence, marking the onset of functional specialization. In lycophytes, monilophytes, and gymnosperms, CER3 remained in single or low copies, with its function uncharacterized. In angiosperms, CER3 copy number expanded, yet core wax biosynthesis remains mediated by one or a few dominant paralogs, while additional copies contribute to tissue specialization and adaptation, reflecting a balance between conservation and innovation (Figure 2A).

From an evolutionary standpoint, the ancestral CER1/3-like gene may have originated from the fusion event of a WAX domain-containing protein and an ERG3/FAH domain-containing protein, and this gene fusion event occurred earlier than the differentiation of all major green plant lineages. Proteins containing only the WAX2 domain have been identified in bacteria and archaea, whereas proteins containing both domains occur in cryptophytes and diatoms [30]. Phylogenomic analyses further suggest that a CER1/3-like gene containing both domains had already emerged before the divergence of Chlorophyta and Streptophyta and was likely retained as a single-copy gene in early green plants. This ancestral protein is hypothesized to have possessed hydrocarbon-producing capability, although its precise biochemical function remains unresolved. Interestingly, CER1/3 homologs were subsequently lost in core Chlorophyta [39]. Previous phylogenomic and molecular clock analyses have estimated that a duplication event of the CER1/3 gene occurred approximately 450–500 million years ago in the common ancestor of land plants, giving rise to the CER1 and CER3 paralogous lineages [30,38]. Our CER3-specific WAX2-domain phylogeny (Figure 2B) does not by itself date this event, as it contains only CER3 orthologs, but its topology—with early-diverging algal sequences at the base and progressively derived land-plant clades—is fully congruent with this hypothesized evolutionary scenario. In the phylogenetic tree, the CER3 homolog from *Pinus pinaster* (PPI00062714) formed a sister branch with the homolog from the green alga *Mesotaenium endlicherianum*, rather than clustering with other gymnosperm sequences (Figure 2B). This topology may reflect the retention of ancestral features in this copy, which might have escaped the lineage-specific duplication and functional diversification observed in other Pinus species, resulting in higher sequence similarity to the algal homolog. However, given the relatively long branch length of this sequence, its phylogenetic position may be affected by long-branch attraction (LBA). Therefore, this topology should not be interpreted as evidence of a direct evolutionary relationship with green algae, but rather as a possible consequence of phylogenetic uncertainty. Additional Pinus genomic resources will be required to further validate this hypothesis.

Based on this topology and previous functional reports, we propose an integrated evolutionary model (Figure 2A) to illustrate the putative trajectory of CER3 functional diversification. In bryophytes, the moss Physcomitrium patens contains at least one CER1 and one CER3 homolog. Both the liverwort Marchantia polymorpha and the hornwort Anthoceros angustus exhibit CER1 and CER3 divergence, although the distinction between CER1 and CER3 homologs remains ambiguous in several early-diverging bryophyte lineages [30,38]. During the fern and gymnosperm stages, CER3 remained in a single-copy or low-copy state, and fern and gymnosperm CER3 still contained a predicted SDR-like module, indicating high structural conservation [30]. Representative ferns include Salvinia cucullata, Azolla filiculoides, and Lygodium japonicum [30]. In gymnosperms, genome analyses of Ginkgo biloba, Picea sitchensis, and Taxus baccata all show CER3 existing as single or low copies (Figure 2) [30].

In angiosperms, the copy number of CER3 expanded, but the core function under normal conditions was still predominantly mediated by a single or a few major genes. Arabidopsis CER3 exists as a single copy, co-expressed with CER1 to form a functional complex participating in alkane synthesis. Likewise, among the three CER3 homologs identified in rice, only OsCER3a plays a predominant role in cuticular wax biosynthesis, whereas the remaining paralogs appear to perform minor or specialized functions and exhibit distinct expression patterns and stress responses [40,41]. Similarly, Quercus mongolica possesses a single-copy CER3 despite substantial expansion of the CER1 gene family [28]. Taken together, the evolutionary history of CER3 in angiosperms is characterized by a balance between strong functional conservation and lineage-specific diversification. In most species, one or a few dominant paralogs maintain the core functions of wax biosynthesis, whereas auxiliary copies contribute to tissue specialization and environmental adaptation, highlighting the coexistence of evolutionary conservation and functional innovation within the CER3 family.

## 3. Multiple Regulatory Mechanisms Governing CER3

**Figure 3:**
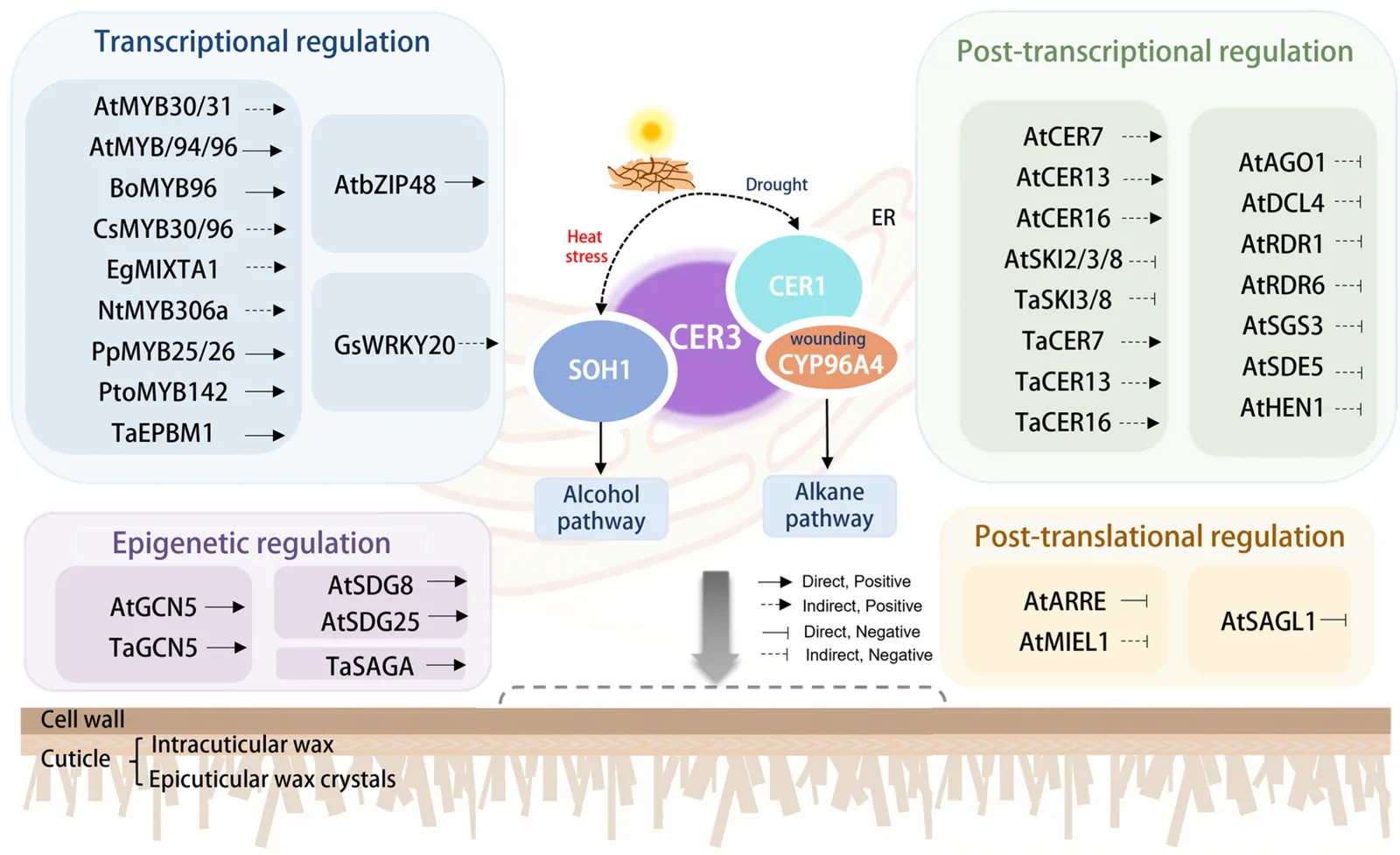
A schematic diagram of the multi-level regulatory network of CER3. This diagram summarizes the regulatory factors involved in the transcriptional, epigenetic, post-transcriptional, and post-translational regulation of *CER3* expression and activity in plants. Regulatory signals originating from these four levels converge upon CER3, which subsequently governs metabolic flux and channels it into the alkane pathway (driven by CER1) or the primary alcohol pathway (driven by SOH1). Arrow types indicate the nature of regulation: solid arrows with filled arrowheads represent direct positive regulation; dashed arrows with filled arrowheads represent indirect positive regulation; solid lines with flat arrowheads represent direct negative regulation; dashed lines with flat arrowheads represent indirect negative regulation. The prefixes denote the following species: *At*, *Arabidopsis thaliana* (Arabidopsis); *Pp*, *Prunus persica* (peach); *Cs*, *Citrus sinensis* (sweet orange); *Bo*, *Brassica oleracea* (kale); *Nt*, *Nicotiana tabacum* (tobacco); *Pto*, *Populus tomentosa* (Chinese white poplar); *Gs*, *Glycine soja* (wild soybean); *Ta*, *Triticum aestivum* (wheat).

*CER3* displays distinct expression patterns in response to endogenous and environmental cues. Among different organs, CER3 is highly expressed in elongating top stems, where wax is actively produced [4]. In contrast, expression data curated in TAIR indicate that CER3 is weakly expressed in roots. In addition, *CER3* is also induced by various stresses including drought, cold, salt, ABA, etc. [17,41,42], but is suppressed by high humidity conditions [43]. The diverse expression patterns of *CER3* reflect its multi-layered regulation, spanning transcriptional, post-transcriptional, post-translational, epigenetic, and protein–protein interaction levels (Figure 3). Transcriptional initiation is the core rate-limiting step of gene expression and the central regulatory hub of the biological process, which requires thorough interpretation to clarify the overall regulatory framework. Transcriptional regulation has abundant published mechanistic studies and sufficient experimental verification data, while research progress and functional evidence for the other three regulatory modes in our research system are currently limited.

### 3.1. Transcriptional Regulation

Until now, several transcription factors, including MYB, WRKY, and basic leucine zipper (bZIP) family members, have been implicated in the regulation of CER3 expression during wax biosynthesis across various organs and environmental conditions [44]. MYB transcription factors have been extensively characterized as conserved regulators of *CER3* expression across diverse organs [45]. MYB94 and MYB96 positively regulate *CER3* expression and wax biosynthesis, and exhibit partially redundant functions in leaves and stems [46,47]. AtMYB30 is involved in the regulation of wax biosynthesis and promotes alkane accumulation in leaves, and may modulate *CER3* expression indirectly through the VLCFAs biosynthetic pathway [48]. Similar regulatory mechanisms appear to be evolutionarily conserved in other species, such as *Nicotiana tabacum* NtMYB306a and *Eustoma grandiflorum* EgMIXTA1 [49,50]. In *Populus tomentosa*, PtoMYB142 indirectly elevates *CER3* transcript abundance via directly activating *CER4* and *KCS6* to boost cuticular wax biosynthesis and related metabolic feedback, rather than through direct transcriptional regulation between *CER4*/*KCS6* and *CER3*, suggesting an evolutionarily conserved yet mechanistically distinct regulatory strategy [51].

In reproductive organs, several MYB genes are identified to participate in the regulation of wax production by affecting *CER3* expression. AtMYB31 directly activates *CER3* expression in floral and silique epidermal tissues, and loss of AtMYB31 markedly reduces wax accumulation, including C29 alkanes, primary alcohols, and total wax in flowers and siliques, while having limited effects in vegetative organs [52]. Likewise, *Prunus persica* PpMYB25/PpMYB26 and Citrus CsMYB96 positively regulate wax deposition and are associated with *CER3* expression changes in fruit exocarp and peel, respectively [53,54]. By contrast, the transcriptional regulatory network controlling *CER3* expression in roots remains largely unknown, despite increasing evidence that root-associated waxes and suberin-derived aliphatics contribute substantially to root development and environmental adaptation.

*CER3* expression is also dynamically modulated by multiple stress-responsive transcription factors in response to environmental cues, including drought, cold and other stresses. Drought is well known to induce wax production. Under drought conditions, MYB96 acts as a central downstream regulator and forms the classical ABA-MYB96-CER3 regulatory module [46,47]. In this module, drought-induced ABA activates MYB96, which then regulates the transcription of wax biosynthesis genes, including *CER3*, thereby promoting alkane formation. CER3 functions in the alkane-forming pathway to produce alkanes, which are major cuticular wax components. This ABA-MYB96-wax biosynthesis cascade thus promotes cuticular barrier formation and reduces water loss. MYB94 is identified to act redundantly with MYB96 to ensure robust wax accumulation under drought. Additionally, Arabidopsis BASIC LEUCINE ZIPPER 48 (bZIP48) is also involved in this regulatory process. It interacts with MYB96, thereby enhancing their respective DNA-binding activities and synergistically activating downstream wax biosynthetic genes, such as *KCSs* and *CER3* [41]. Besides Arabidopsis, the regulatory roles of MYB96 have also been identified in *Brassica oleracea*. Drought-induced ABSCISIC ACID-RESPONSIVE ELEMENT BINDING FACTOR 2 (*BoABF2*) directly activates *BoMYB96*, which subsequently regulates *BoCER3* expression and promotes wax accumulation, particularly increasing the levels of C29 alkanes, C29 secondary alcohols, C29 ketones, and C42 wax esters [55]. In citrus, CsMYB30 positively regulates *CER3* expression during water deficit, contributing to enhanced drought tolerance [56]. Cold stress also induces wax production. Wax accumulation serves as a critical adaptive strategy for plants to cope with cold stress, contributing to enhanced membrane stability and water retention. Under cold conditions, CER3-associated wax biosynthesis is also regulated by MYB96, promoting alkane accumulation and strengthening the cuticular barrier [41]. In addition to MYB proteins, members of the WRKY family participate in environmental regulation of CER3 [57]. Given that drought stress induces *Glycine soja GsWRKY20* expression and its overexpression up-regulates CER3 and activates wax biosynthesis, we propose that GsWRKY20 may function as a positive regulator of cuticular wax accumulation under drought stress [58]. Collectively, these findings indicate that CER3 serves as an important transcriptional integration hub where developmental programs and environmental signals converge to dynamically regulate wax biosynthesis and optimize plant adaptation to changing environments.

### 3.2. Post-Transcriptional Regulation

Primary transcripts must undergo extensive processing before becoming mature mRNAs, whereas aberrant transcripts are rapidly eliminated by RNA surveillance pathways [59,60,61]. Beyond RNA maturation, RNA degradation pathways prevent the accumulation of defective transcripts, suppress inappropriate activation of post-transcriptional gene silencing (PTGS), and contribute to environmental adaptation through coordinated nuclear and cytoplasmic RNA turnover [62,63,64]. The RNA exosome is a central component of the RNA quality-control machinery. Composed of highly conserved core subunits and accessory factors, the exosome cooperates with the cytoplasmic SKI complex (SKI2, SKI3, and SKI8) and SKI7 to mediate 3′–5′degradation of aberrant RNAs [65,66,67]. In Arabidopsis, *CER3* is regulated via well-documented exosome-mediated RNA quality control. *CER3* transcript accumulation relies on an RNA surveillance module composed of CER7 (RRP45B), CER16 (RIPR), RST1 and the SKI complex. This protein complex degrades abnormal transcripts and suppresses the PTGS pathway mediated by RDR1/RDR6, SGS3, DCL4 and AGO1; such repression ultimately stabilizes CER3 mRNA levels.

CER7, as a component of the exosome complex, does not directly regulate *CER3* transcripts. Instead, CER7 preserves *CER3* expression by degrading aberrant RNAs that would otherwise enter the PTGS pathway and generate CER3-targeting siRNAs [68]. Properly polyadenylated mRNAs are shielded from exosomal decay by cytoplasmic cap-binding and poly(A)-binding protein complexes. Aberrant *CER3* transcripts generated upon CER7 loss likely possess incomplete or absent poly(A) tails; this feature disrupts protective ribonucleoprotein assembly, exposing the RNA 3′ terminus for 3′→5′ exoribonucleolytic digestion by the exosome core complex. When CER7 function is disrupted, aberrant RNAs accumulate and trigger PTGS-mediated production of CER3-targeting siRNAs, ultimately resulting in *CER3* silencing [69]. In this process, CER16 serves as a key inhibitory factor that maintains stable *CER3* expression by blocking the PTGS pathway. In cer16 mutants, endogenous small interfering RNAs (siRNAs) targeting *CER3* accumulate in large quantities, leading to *CER3* silencing and significantly reduced alkane synthesis [69]. This PTGS pathway strictly depends on core RNA silencing components such as RDR1, RDR6, SGS3, AGO1, and DCL4, which jointly participate in generating small RNAs targeting CER3 (including trans-acting small interfering RNAs, tasiRNAs), directly mediating *CER3* transcript degradation [70,71]. Simultaneous mutation of RDR1, RDR6, SGS3, AGO1, or DCL4 can relieve *CER3* silencing and restore wax synthesis [71]. Additionally, the SKI complex synergizes with CER7 in *CER3* mRNA turnover and quality control [72]. RST1 and CER16 serve as molecular bridges connecting the exosome core to the SKI complex, jointly forming an RNA surveillance-defense system that prevents abnormal mRNA accumulation and non-specific gene silencing [73]. This series of studies reveals that *CER3* expression levels are maintained in a dynamic equilibrium involving the RNA exosome, SKI complex, 5′–3′ EXORIBONUCLEASE 4 (XRN4), and the PTGS machinery [74].

This regulatory mechanism is also conserved in monocots. In Triticum aestivum, TaCER7, TaCER13, TaCER16 and the SKI complex synergistically maintain *TaCER3* mRNA stability. The cap-binding complex (CBC), TaSERRATE (C2H2 zinc-finger protein) and Elongator also participate in its regulation [75]. This indicates that from Arabidopsis to wheat, *CER3* is under strict surveillance by the RNA quality control system. Nevertheless, the structural basis for the high sensitivity of *CER3* transcripts to RNA quality control remains unclear, and whether there is a clear hierarchical relationship among the RNA exosome, CBC and PTGS lacks direct evidence.

### 3.3. Post-Translational Regulation

Post-translational regulatory mechanisms include ubiquitination, phosphorylation, and SUMOylation. To date, only the ubiquitin-mediated regulation of CER3 has been experimentally validated. Ubiquitination is a widespread post-translational modification in eukaryotes in which ubiquitin molecules are covalently attached to lysine residues of target proteins through the sequential actions of E1 ubiquitin-activating enzymes, E2 ubiquitin-conjugating enzymes, and E3 ubiquitin ligases, thereby marking substrates for degradation by the 26S proteasome [76,77]. Owing to their substrate specificity, E3 ubiquitin ligases play a central role in determining target recognition and have emerged as key regulators of protein abundance in response to developmental and environmental cues. Several E3 ubiquitin ligases have been implicated in the post-translational regulation of CER3 through ubiquitin-mediated protein degradation. Among them, the F-box protein SMALL ANDGLOSSY LEAVES1 (SAGL1) is currently the best-characterized negative regulator of CER3 under high humidity. SAGL1 is induced by high humidity, and its protein products remain stable under such conditions. SAGL1 directly recognizes and ubiquitinates CER3, promoting its proteasomal degradation and consequently suppressing the biosynthesis of fatty acids, primary alcohols, and alkanes [43].

Another important negative regulator of CER3 is ABA-related RING-type E3 ligase (ARRE), which is highly expressed in mature stem internodes and leaves. ARRE promotes CER3 degradation and contributes to the developmental termination of wax biosynthesis during tissue maturation [78]. AtARRE appears to act predominantly in aerial tissues, consistent with its expression profiles revealed by qRT-PCR quantification and AtARRE: GUS histochemical staining assays. AtARRE exhibits abundant transcript accumulation and prominent promoter activity in rosette leaves, cauline leaves, floral organs and siliques; comparatively weak expression was detected in roots, and stem epidermal expression was confined to aged basal internodes rather than young elongating stem regions. In addition to direct regulation of CER3 protein stability, some E3 ligases influence CER3 expression indirectly. For example, MYB30-INTERACTING E3 LIGASE 1 (MIEL1) suppresses CER3 transcription through ubiquitin-mediated degradation of the transcription factors MYB96 and MYB30 [79]. This regulatory mechanism appears to be evolutionarily conserved, as a similar function has been reported for BoMIEL1 in kale [55]. Furthermore, CER9, an endoplasmic reticulum-localized E3 ubiquitin ligase, has been implicated in maintaining lipid homeostasis through the endoplasmic reticulum-associated degradation (ERAD) pathway. Although no direct interaction between CER9 and CER3 has been reported, CER9 may indirectly influence wax metabolism by modulating lipid metabolic homeostasis [80]. Collectively, these findings highlight ubiquitin-mediated proteolysis as an important layer of CER3 regulation. Whether distinct E3 ubiquitin ligases function sequentially or coordinately during plant development to fine-tune CER3 abundance and wax biosynthesis remains an intriguing possibility that awaits further experimental investigation.

### 3.4. Epigenetic Regulation

Epigenetic modifications constitute an important layer of the regulatory network controlling cuticular wax biosynthesis [26]. Major epigenetic mechanisms include histone acetylation, histone methylation, histone H2B monoubiquitination (H2Bub), and chromatin remodeling. Among these epigenetic mechanisms, histone acetylation represents one of the best-characterized pathways regulating *CER3* transcription. Histone acetylation is catalyzed by Histone acetyltransferases (HATs), which generally promote gene transcription by establishing a more open chromatin configuration [81,82]. In Arabidopsis, the histone acetyltransferase GENERAL CONTROL NON-REPRESSED 5 (GCN5) directly associates with the *CER3* promoter and promotes H3K9/H3K14 acetylation, thereby establishing an open chromatin state that facilitates *CER3* transcription [83]. The *gcn5* mutant exhibits a substantial reduction in epicuticular wax crystals, with total wax load decreasing to approximately 63% of wild-type levels. Major wax constituents, including alkanes, ketones, aldehydes, and alcohols, are all significantly reduced and accompanied by decreased *CER3* expression. Importantly, overexpression of *CER3* partially rescues the wax-deficient phenotype of *gcn5*, demonstrating that *CER3* is a key downstream target of the GCN5 regulatory pathway. This regulatory mechanism may be evolutionarily conserved in monocot species. In wheat, TaGCN5 forms a ternary complex with the enoyl-CoA reductase promoter-binding MYB transcription factor 1 (TaEPBM1) and the adaptor protein ALTERATION/DEFICIENCY IN ACTIVATION 2 (TaADA2) to activate *TaECR* expression through histone acetylation [84]. In addition, the TaSAGA complex has been implicated in promoting *TaCER3* expression through acetylation-mediated chromatin regulation [75], further supporting the importance of histone acetylation in wax biosynthesis.

In addition to acetylation, histone methylation also contributes to the fine regulation of *CER3* expression. In Arabidopsis, the histone methyltransferases SET DOMAIN GROUP 8 (SDG8) and SDG25 regulate *CER3* transcription during immune responses by maintaining H3K36 and H3K4 methylation levels. Loss of either SDG8 or SDG25 results in reduced *CER3* expression and increased leaf cuticle permeability [85]. Notably, histone methylation and H2B monoubiquitination exhibit extensive functional crosstalk in Arabidopsis [86].

Overall, current evidence indicates that histone acetylation and methylation play important roles in regulating *CER3* expression, whereas the contributions of H2B monoubiquitination and chromatin remodeling remain largely indirect. Furthermore, the potential involvement of DNA methylation, higher-order chromatin architecture, and repressive histone modifications in *CER3* regulation remains largely unexplored and warrants further investigation.

### 3.5. CER3 Activity Is Modulated Through Its Assembly into Protein Complexes with Interacting Partners

Besides the above-mentioned regulation mechanism, CER3 activity is also regulated by forming complexes with other proteins. Until now, CER3 has been identified to interact with CER1, SOH1 and CYP96A4 for channeling the carbon resource into the wax or cutin synthesis pathway in response to environmental cues.

#### 3.5.1. Coordinated Action of CER3, CER1 Homologs and Cytochrome B5

CER1 is a key component of the cuticular wax alkane-forming pathway and is considered to be responsible for the conversion of very-long-chain aldehydes into alkanes. Yeast two-hybrid assays together with co-expression and co-purification analyses demonstrated direct interaction between CER1 and CER3. In addition, Cytochrome b5 (CYTB5) is also associated with the CER1–CER3 complex. However, it does not serve as the catalytic core but rather functions as an essential cofactor and electron shuttle that ensures efficient CER1-mediated reduction during cuticular wax biosynthesis by supplying reducing equivalents and stabilizing the protein complex [22]. Functional reconstruction experiments further showed that CER3 converts very-long-chain acyl-CoAs into aldehyde intermediates, whereas CER1 utilizes these intermediates for alkane production, indicating that the two proteins operate as a tightly coupled functional unit during catalysis [22]. The biological significance of the CER1-CER3 interaction lies primarily in regulating alkane biosynthetic efficiency and product specificity.

CER1-LIKE1, a homolog of Arabidopsis CER1, can also interact with CER3 and CYTB5 to form a functional complex. However, CER1-LIKE1 displays a preference for shorter-chain substrates, resulting predominantly in the production of C25-C29 alkanes, whereas the CER1-CER3 complex preferentially generates C29-C31 alkanes [30,38,87]. In specific tissues such as floral organs, CER1-LIKE1 can partially substitute for CER1 during complex assembly, thereby altering alkane composition and contributing to tissue-specific wax deposition. These findings indicate that CER1 and its homologs not only serve as the principal catalytic partner of CER3 but also play an important role in determining alkane chain-length distribution and tissue-specific wax accumulation.

#### 3.5.2. Coordinated Action Between CER3 and SOH1 Homologs

Besides CER1, CER3 also interacts with SOH1 and its homolog SOH1-LIKE. SOH1 and its homolog were recently identified as key aldehyde reductases (AHRs) responsible for primary alcohol biosynthesis in Arabidopsis cuticular waxes [25]. The classical model demonstrates that fatty acyl-CoA reductases (FARs, including CER4) directly convert very-long-chain acyl-CoAs into primary alcohols, with no free aldehyde intermediates released in the reaction pathway [88,89]. However, recent studies revealed the existence of an alternative two-step reduction pathway in Arabidopsis leaves. In this pathway, CER3 likely participates in the reduction of very-long-chain acyl-CoAs to aldehydes, which are subsequently reduced to primary alcohols by SOH1 or SOH1-LIKE [25]. Genetic analyses demonstrated that SOH1 and SOH1-LIKE are the major contributors to C30 and C32 primary alcohol biosynthesis in Arabidopsis leaves, establishing them as critical partners of CER3. Despite their partially redundant functions, SOH1 and SOH1-LIKE exhibit distinct expression patterns and catalytic properties. SOH1 is predominantly expressed in leaves and is strongly induced by elevated temperature, whereas SOH1-LIKE shows broader but generally lower expression and weaker heat responsiveness [25]. Moreover, SOH1 preferentially contributes to C30 primary alcohol production, while SOH1-LIKE displays relatively higher activity toward C32 substrates. Consistently, the two single mutants exhibit distinct alterations in primary alcohol chain-length composition, and the double mutant shows a much stronger reduction in total primary alcohol accumulation [25]. These findings indicate that SOH1 and SOH1-LIKE function cooperatively but have undergone partial subfunctionalization in both transcriptional regulation and substrate preference.

The interaction between CER3 and SOH1 is supported by multiple lines of physical, biochemical, and genetic evidence. Luciferase complementation imaging (LCI) and split-ubiquitin yeast two-hybrid (SuY2H) assays demonstrated direct interactions between CER3 and both SOH1 proteins, with all proteins co-localizing to the endoplasmic reticulum [25]. Heterologous reconstruction in yeast further showed that expression of either CER3 or SOH1 alone failed to produce primary alcohols, whereas co-expression of both proteins resulted in the accumulation of C26–C30 primary alcohols. Moreover, overexpression of SOH1 in the *cer3* mutant background was unable to restore alcohol production. Collectively, these results demonstrate that CER3 and SOH1 constitute a functional primary alcohol biosynthetic module.

The most significant biological implication of the CER3-SOH1 interaction is its role in regulating wax metabolic flux allocation. Both CER1 and SOH1 competitively interact with CER3 [25]. Association of CER3 with CER1 directs metabolism toward alkane production, whereas interaction with SOH1 or SOH1-LIKE promotes primary alcohol biosynthesis. Competitive interaction assays demonstrated that overexpression of CER1 weakens the CER3-SOH1 interaction signal and vice versa. Thus, CER3 is proposed to function as a metabolic flux hub controlling wax composition. Moreover, environmental cues influence this process. Drought stress strongly induces CER1 expression and promotes alkane accumulation, thereby enhancing cuticular hydrophobicity and reducing non-stomatal water loss [90,91,92]. In contrast, elevated temperature stimulates SOH1 expression and favors primary alcohol accumulation [25]. Consequently, environmental cues may modulate the selective interactions of CER3 with CER1 or SOH1, thereby directing carbon flux toward specific wax biosynthetic pathways. Notably, the CER3-SOH1 module appears to be evolutionarily conserved across land plants. Phylogenetic analyses revealed the widespread presence of orthologous *CER3* and *CER1* genes in bryophytes, monocots, and dicots [30]. SOH1 homologs from rice and Physcomitrium patens are capable of interacting with Arabidopsis CER3 and cooperatively producing primary alcohols in yeast expression systems [25], indicating that this module has been maintained through long-term evolutionary selection during land plant adaptation. Moreover, phylogenetic analyses indicate that SOH1-LIKE homologs are also widely distributed in angiosperms, although their copy number and sequence diversification appear greater than those of SOH1, suggesting that subfunctionalization of the SOH1 family may have contributed to the diversification of primary alcohol biosynthesis during plant evolution.

#### 3.5.3. Coordinated Action Between CER3 and CYP96A4

Mechanical wounding triggers extensive remodeling of epidermal lipids to rapidly restore barrier integrity. Following tissue damage, the total amounts of both cuticular waxes and cutin monomers increase substantially, accompanied by pronounced alterations in wax composition, including enhanced accumulation of alkanes and other hydrophobic constituents [93,94]. These metabolic changes are associated with strong transcriptional induction of multiple wax biosynthetic genes, including CER1, CER3, and CYP96A4, as well as genes involved in cutin formation and jasmonic acid (JA) signaling. The coordinated induction of these genes suggests that CER3-containing complexes participate not only in constitutive wax biosynthesis but also in wound-induced epidermal lipid remodeling.

Table 1 provides a summary of reported regulators and interacting partners of CER3. This table summarizes all known transcriptional, epigenetic, post-transcriptional, post-translational regulators, and protein interaction partners that modulate CER3 expression or protein activity. Evidence types are categorized as follows: Biochemical (e.g., protein–protein interaction, ubiquitination); Transcriptional/Chromatin (e.g., promoter binding, ChIP, histone modification); Genetic (e.g., mutant phenotype, overexpression); or Expression (e.g., co-expression correlation). “Direct” indicates that the regulator physically binds to the CER3 promoter, protein, or transcript; “Indirect” indicates that the effect is mediated through an intermediate factor or metabolic feedback.

CYP96A4 is an endoplasmic-reticulum-localized cytochrome P450 monooxygenase whose expression is strongly induced by mechanical wounding and JA signaling. It contributes to the accumulation of cuticular waxes and cutin monomers during wound-induced epidermal barrier repair [93,94]. Unlike CER1 and SOH1, which directly participate in wax biosynthesis, CYP96A4 acts as a cofactor of the CER1/CER3 alkane-forming complex and may also modulate JA signaling. CYP96A4 physically interacts with both CER3 and CER1. Loss of CYP96A4 significantly impairs wound-induced accumulation of waxes and cutin monomers [94], indicating that CYP96A4 is required for the full wound-induced accumulation of wax and cutin monomers. Furthermore, disruption of CYP96A4 results in reduced expression of JA-responsive genes, highlighting its close relationship with wound signaling pathways. The biological significance of the CER3-CYP96A4 interaction lies in linking environmental signaling with wax biosynthesis. Following mechanical damage, JA signaling induces *CYP96A4* expression, and CYP96A4 interacts with CER3 and primarily influences CER3-mediated aldehyde production, which may contribute to wound-induced wax biosynthesis and restoration of epidermal barrier integrity. In contrast to SOH1, which regulates metabolic flux through competition for CER3 binding, CYP96A4 is more likely to function as a stress-responsive regulatory component involved in the dynamic reorganization of CER3-associated complexes. This observation further emphasizes that CER3 serves not only as a catalytic component of wax biosynthesis but also as an integrative platform coordinating adaptive responses to environmental challenges.

Collectively, the interactions between CER3 and its partners considerably expand the functional scope of CER3-containing metabolic complexes. A comprehensive summary of all CER3 regulators and interacting partners is provided in Table 1. Through subunit replacement by CER1-LIKE1, competitive recruitment of SOH1, and stress-induced association with CYP96A4, CER3-containing complexes exhibit remarkable functional plasticity and regulatory flexibility, enabling plants to dynamically coordinate wax biosynthesis, carbon flux allocation, wound repair, and environmental adaptation.

## 4. Conclusions

Understanding the molecular function and regulatory mechanism of CER3 is critical for revealing how plants establish and modify cuticular wax barriers during evolution and environmental adaptation. As the key component connecting wax biosynthesis with environmental responses, CER3 provides an important framework for investigating the regulation of epidermal barrier formation and its contribution to plant stress adaptation.

From an applied perspective, manipulation of CER3-mediated wax biosynthesis represents a potential strategy for improving crop resilience. Altering CER3 expression or its regulatory networks may modify wax accumulation and composition, thereby affecting water retention, pathogen resistance, and postharvest performance. In cereal crops such as wheat and rice, enhanced wax deposition associated with CER3 activity has been linked to reduced water loss and improved adaptation. In horticultural crops, the CER3-mediated wax biosynthesis pathway contributes to the formation and composition of fruit cuticular wax, thereby influencing cuticle integrity and associated physiological properties [95,96]. Functional CER3 activity promotes the accumulation of intact wax layers, which can reduce fruit water loss, delay postharvest deterioration, and enhance resistance to fungal infection, thereby contributing to the maintenance of fruit quality during storage in tomato, apple, mango, and other climacteric fruits [97,98]. Modulation of the CER3 regulatory module through genetic approaches may provide a potential strategy for optimizing endogenous wax deposition and improving postharvest performance in horticultural crops.

Despite substantial progress, many aspects of CER3 regulation remain unresolved. The mechanisms underlying tissue-specific regulation, lineage-specific diversification, and the integration of transcriptional, post-transcriptional, post-translational and epigenetic regulatory pathways are still poorly understood across plant species. Future studies combining comparative genomics, multi-omics approaches, protein interactome analyses, and genome-editing technologies will be essential to establish a comprehensive understanding of CER3 regulatory networks. Such efforts will clarify how CER3 coordinates environmental signals with wax metabolism and will further reveal the evolutionary and functional principles underlying plant epidermal barrier formation. A deeper understanding of CER3 biology will advance our knowledge of plant lipid metabolism and provide valuable molecular targets for developing crops with improved stress resilience and agronomic performance.

## Figures and Tables

**Table 1:** Summary of reported regulators and interacting partners of CER3.

Number	Gegulator	Species	Organ	Condition	Type of Evidence	Direct/Indirect	Effect on CER3	Reference
1	AtMYB96	*Arabidopsis thaliana*	Leaves/aerial organs	Drought/ABA/cold	EMSA; ChIP-qPCR; transactivation assay	Direct	Positive	[41,47]
2	AtbZiP48	*Arabidopsis thaliana*	Leaves	Cold	Y1H; EMSA; ChIP-qPCR; transactivation assay	Direct	Positive	[41]
3	AtMYB94	*Arabidopsis thaliana*	Leaves/aerial organs	Drought/ABA	ChIP-qPCR; transactivation assay	Direct	Positive	[46,47]
4	AtMYB30	*Arabidopsis thaliana*	Leaves	Pathogen infection/HR	Expression analysis; transactivation assay	Indirect	Positive	[48]
5	NtMYB306a	*Nicotiana tabacum*	Leaves/trichomes	Normal growth; SA/cold	Expression analysis; overexpression analysis	Indirect	Positive	[49]
6	EgMIXTA1	*Eustoma grandiflorum*	Leaves	Normal growth	Expression analysis; overexpression analysis	Indirect	Positive	[50]
7	PtoMYB142	*Populus tomentosa*	Leaves	Drought	Transactivation assay; promoter analysis	Indirect	Positive	[51]
8	AtMYB31	*Arabidopsis thaliana*	Flowers, siliques, seeds	Reproductive development	Transactivation assay; expression analysis	Indirect	Positive	[52]
9	PpMYB25/26	*Prunus persica*	Fruit exocarp	Fruit development	Y1H; transactivation assay	Direct	Positive	[53]
10	TaEPBM1	*Triticum aestivum*	Leaves	Normal growth	Y1H; ChIP-qPCR; transactivation assay	Direct	Positive	[75]
11	CsMYB96	*Citrus sinensis*	Fruit peel	Postharvest water loss	Expression analysis; overexpression analysis	Indirect	Positive	[54]
12	BoMYB96	*Brassica oleracea* var. *acephala*	Leaves	Drought	Y1H; transactivation assay	Direct	Positive	[55]
13	CsMYB30	*Citrus sinensis*	Leaves	Salt/drought	Expression analysis; heterologous overexpression	Indirect	Positive	[56]
14	GsWRKY20	*Glycine soja*/*Arabidopsis thaliana*	Leaves	Normal growth	Expression analysis; heterologous overexpression	Indirect	Positive	[58]
15	AtCER7 (AtRRP45b)	*Arabidopsis thaliana*	Stems	Normal growth	Genetic analysis; CER3 expression; small-RNA analysis	Indirect	Positive	[68]
16	AtCER16 (ATRIPR)	*Arabidopsis thaliana*	Stems	Normal growth	Genetic analysis; RNA analysis; protein interaction	Indirect	Positive	[69,73]
17	AtCER13 (AtRST1)	*Arabidopsis thaliana*	Stems	Normal growth	Genetic analysis; RNA analysis; protein interaction	Indirect	Positive	[73]
18	AtRDR1	*Arabidopsis thaliana*	Stems	Stem development	Genetic suppressor analysis; CER3 expression; tasiRNA analysis	Indirect	Negative (*cer7*)	[70,71]
19	AtRDR6	*Arabidopsis thaliana*	Stems	Stem development	Genetic suppressor analysis; CER3 expression; tasiRNA analysis	Indirect	Negative (*cer7*)	[71]
20	AtSGS3	*Arabidopsis thaliana*	Stems	Stem development	Genetic suppressor analysis; CER3 expression; tasiRNA analysis	Indirect	Negative (*cer7*)	[70,71]
21	AtSDE5	*Arabidopsis thaliana*	Stems	Stem development	Genetic suppressor analysis; CER3 expression; tasiRNA analysis	Indirect	Negative (*cer7*)	[70,71]
22	AtDCL4	*Arabidopsis thaliana*	Stems	Stem development	Genetic suppressor analysis; CER3 expression; tasiRNA analysis	Indirect	Negative (*cer7*)	[70,71]
23	AtAGO1	*Arabidopsis thaliana*	Stems	Stem development	Genetic suppressor analysis; CER3 expression; tasiRNA analysis	Indirect	Negative (*cer7*)	[70,71]
24	AtHEN1	*Arabidopsis thaliana*	Stems	Stem development	Genetic analysis; CER3 expression; tasiRNA analysis	Indirect	Negative (*cer7*)	[70,71]
25	AtSKI2/3/8	*Arabidopsis thaliana*	Stems	Stem development	Genetic suppressor analysis; CER3 expression; tasiRNA analysis	Indirect	Positive(*cer7*)	[72]
26	AtGCN5	*Arabidopsis thaliana*	Stems	Normal growth	RNA-seq; ChIP-qPCR; histone acetylation analysis; genetic complementation	Direct	Positive	[83]
27	AtSDG8/25	*Arabidopsis thaliana*	Leaves	Mock or B. cinerea inoculation	Gene expression, ChIP, Mutant phenotype	Direct	Positive	[85]
28	TaGCN5	*Triticum aestivum*	Leaves	Normal growth	VIGS; ChIP-qPCR; histone acetylation analysis	Direct	Positive (H3K9/K14ac)	[75]
29	TaADA2	*Triticum aestivum*	Leaves	Normal growth	VIGS; ChIP-qPCR; histone acetylation analysis	Direct	Positive (H3 acetylation)	[75]
30	TaSCS1A/TaSCS1B/TaSCS2	*Triticum aestivum*	Leaves	Normal growth	VIGS; ChIP-qPCR; histone acetylation analysis	Direct	Positive (redundant)	[75]
31	TaCER7	*Triticum aestivum*	Leaves	Normal growth	VIGS; CER3 expression analysis	Indirect	Positively	[75]
32	TaCER13	*Triticum aestivum*	Leaves	Normal growth	VIGS; CER3 expression analysis	Indirect	Positively	[75]
33	TaCER16	*Triticum aestivum*	Leaves	Normal growth	VIGS; CER3 expression analysis	Indirect	Positively	[75]
34	AtARRE	*Arabidopsis thaliana*	Leaves and stems	Pathogen exposure	Protein interaction; degradation assay	Direct	Negative (post-translational)	[78]
35	AtMIEL1	*Arabidopsis thaliana*	Stems	Pathogen exposure	Genetic analysis; CER3 expression analysis	Indirect	Negative (post-translational)	[79]
36	AtSAGL1	*Arabidopsis thaliana*	Leaves, stems and roots	High humidity	Protein interaction; degradation assay	Direct	Negative (post-translational)	[43]

## Data Availability

All protein sequences used for the phylogenetic analysis in this study are available from publicly accessible databases, including Phytozome, NCBI Genome, Ensembl Plants, JGI Genome Portal, and Gymno PLAZA 1.0, as detailed in the figure caption.

## Supplementary Materials

The following supporting information can be downloaded at https://www.mdpi.com/article/10.3390/biology15161437/s1, Table S1: Accession numbers of proteins used for phylogenetic tree construction and corresponding information.
